# Patterns of Cardiomyopathy in Patients Presenting to a Tertiary Care Hospital

**DOI:** 10.7759/cureus.80794

**Published:** 2025-03-18

**Authors:** Ikram Ullah, Sher W Khan, Ayesha Fayyaz, Kamran Khan, Farooq Ahmad, Sayeeda M Shah

**Affiliations:** 1 Cardiology, Northwest General Hospital and Research Centre, Peshawar, PAK; 2 Adult Cardiology, Lady Reading Hospital and Medical Teaching Institute, Peshawar, PAK; 3 Internal Medicine, Community Health Connections, Fitchburg, USA; 4 Cardiology, Lady Reading Hospital and Medical Teaching Institute, Peshawar, PAK; 5 Cardiology, Khyber Teaching Hospital, Peshawar, PAK; 6 Obstetrics and Gynecology, Khyber Teaching Hospital, Peshawar, PAK

**Keywords:** cardiomyopathy, epidemiology, family history, gender distribution, ischemic cardiomyopathy, peshawar, restrictive cardiomyopathy, tertiary care hospital

## Abstract

Background: Cardiomyopathy is a broad category of myocardial conditions that have a substantial effect on heart function. Improving patient treatment requires a knowledge of its epidemiology.

Objective: The aim of this study was to determine the pattern of cardiomyopathy in patients presenting to a tertiary care hospital in Peshawar, Pakistan.

Methodology: This cross-sectional study was conducted at the Department of Cardiology, Northwest General Hospital & Research Centre, Peshawar, from December 14, 2022, to June 14, 2023. There were 79 individuals with cardiomyopathy who were 16 years of age or older. Clinical and demographic information, such as age, gender, BMI, length of illness, and family history, were gathered. Cardiomyopathy patterns were classified using echocardiographic evaluations, and IBM SPSS Statistics for Windows, version 25 (IBM Corp., Armonk, NY) was employed for statistical analysis.

Results: The average age of the 79 participants was 45.72 ± 2.45 years, and 40.5% (n=32) were between the ages of 51 and 60. There were 63.3% male individuals (n=50) and 36.7% female individuals (n=29). With 69.6% (n=55) and 30.4% (n=24) having a duration of symptoms ≤1 month and >1 month, respectively. 38.0% (n=30) had a family history of cardiomyopathy. With dilated, hypertrophic, and peripartum cardiomyopathy each at 15.2%, the most prevalent forms of cardiomyopathy were restrictive (20.3%, n=16), ischemic (17.7%, n=14), and arrhythmogenic right ventricular (16.5%, n=13). BMI (p = 0.000) and illness duration (p = 0.000) were substantially correlated with dilated and hypertrophic cardiomyopathies. Older age groups, especially those between the ages of 51 and 60, had a greater prevalence of cardiomyopathy (p = 0.000). Dilated cardiomyopathy (p = 0.000) and peripartum cardiomyopathy (p = 0.000) were significantly influenced by family history.

Conclusion: The research highlights the variety of cardiomyopathy patterns seen in a tertiary care facility, with ischemic and restrictive forms being the most prevalent. This highlights the need for specialized diagnosis and treatment strategies.

## Introduction

Among patients presenting to a tertiary care hospital, cardiomyopathy encompasses a range of myocardial disorders that critically impact cardiac function and clinical outcomes [1]. It is a major contributor to heart failure, arrhythmias, and sudden cardiac death [2]. Dilated, hypertrophic, restricted, and arrhythmogenic right ventricular cardiomyopathy are among the several types of cardiomyopathy that have different pathophysiological causes, clinical manifestations, and prognostic consequences [3]. Enhancing diagnostic precision and enhancing patient care requires an understanding of the epidemiology and pattern of cardiomyopathy [4].

Cardiomyopathy affects approximately one in 500 adults worldwide, with an estimated prevalence of six million cases, and its occurrence varies based on genetic predisposition, coexisting medical conditions, and environmental factors [5-7]. While ischemic heart disease and genetic abnormalities are the predominant reasons in industrialized areas, infectious and dietary factors continue to be important in poorer nations [8]. Cardiomyopathy may manifest in a variety of ways, from accidentally discovered asymptomatic patients to acute heart failure necessitating sophisticated procedures [9].

Social determinants of health, such as economic instability, inadequate healthcare access, and disparities in education, significantly influence cardiovascular outcomes. As highlighted by Borkowski et al. (2024), lower socioeconomic status is strongly associated with higher rates of hypertension, diabetes, and obesity, all of which contribute to worsened cardiomyopathy prognosis [10]. These populations also face systemic barriers such as food and housing insecurity, lack of insurance coverage, and a higher prevalence of mental health disorders, further compounding cardiovascular risk. Timely therapy approaches might possibly change the course of the illness and increase patient survival when cardiomyopathy is identified and classified early [11].

Studies conducted in hospitals provide important information on the clinical and demographic range of cardiomyopathy in certain groups [12]. As referral hubs, tertiary care institutions see a wide variety of patients, which makes them perfect places to research illness trends [13]. The distribution of cardiomyopathy subtypes and frequency in these hospitals mirrors larger patterns in the regional healthcare system. Examining these trends is crucial for guiding future research, allocating resources, and customizing healthcare policy [14,15]. Accurately diagnosing and treating cardiomyopathy still presents difficulties, despite improvements in cardiac imaging, biomarker assessment, and genetic testing. Clinical decision-making is further complicated by variations in illness presentation, characteristics that overlap with other cardiac disorders, and restricted access to expert treatment in places with low resources. Better clinical recommendations and treatment strategies may be developed by systematically analyzing the patterns of cardiomyopathy in tertiary care facilities.

Research objective

The objective of this study was to determine the prevalence and distribution of different cardiomyopathy subtypes among patients presenting to a tertiary care hospital in Peshawar, Pakistan, over a six-month period. Specifically, the study aimed to categorize cardiomyopathy based on demographic factors (age, gender, BMI, and family history) and clinical characteristics (echocardiographic findings and comorbidities). Furthermore, it sought to compare these patterns with global trends and assess the potential influence of genetic predisposition and environmental factors.

## Materials and methods

Study design and setting

This cross-sectional study was conducted at the Department of Cardiology, Northwest General Hospital & Research Centre, Peshawar. The study duration was from December 14, 2022, to June 14, 2023.

Sample size

Accordingly, the WHO sample size calculation was applied to determine an appropriate sample size, factoring in a 95% confidence level, a 6% margin of error, and an estimated 8% prevalence of hypertrophic cardiomyopathy. This yielded an estimated sample of 79 patients, selected through a non-probability sequential sampling approach. The prevalence of cardiomyopathy varies across subtypes and populations, with our estimated 8% prevalence for hypertrophic cardiomyopathy being higher than commonly reported figures. However, previous studies have documented similar or higher rates in specific cohorts. Hypertrophic cardiomyopathy affects approximately 0.2% to 0.5% of the general population [16], while dilated cardiomyopathy occurs in one in 250 adults [17]. Restrictive cardiomyopathy, being relatively rare, accounts for 2% to 5% of all cardiomyopathy cases [3]. These variations underscore the need for region-specific epidemiological data to better assess disease burden.

Inclusion and exclusion criteria

This research included both male and female patients with cardiomyopathies who were 16 years of age or older. Individuals having a history of pericarditis or pericardial effusion, those with myocarditis exhibiting chamber dilatation, and those with congenital heart disorders with or without heart failure were excluded.

Data collection

A consultant cardiologist with at least five years of post-fellowship experience performed the diagnoses using a GE Vivid T8 (GE Healthcare, Chicago, IL) echocardiograph. The echocardiographic assessment incorporated both structural and functional evaluations through two-dimensional (B-mode) and M-mode imaging. Additionally, pulsed-wave and continuous-wave Doppler techniques were used to assess hemodynamic parameters, ensuring a comprehensive analysis of cardiac function.

The evaluation focused on critical parameters, including the early/atrial ventricular filling velocity ratio, chamber geometry, myocardial thickness, and left ventricular ejection fraction (EF%). Fractional shortening was measured to assess myocardial contractility, while the presence of left ventricular outflow tract obstruction was specifically examined for hypertrophic cardiomyopathy. These echocardiographic findings were essential in accurately classifying and diagnosing various cardiomyopathy subtypes.

In addition to echocardiographic findings, baseline demographic and clinical data, including age, gender, BMI, duration of illness, and family history of cardiomyopathy, were systematically recorded using a standardized proforma (Table 1). This structured approach ensured consistency and accuracy in data collection, allowing for a robust analysis of cardiomyopathy patterns and their association with demographic and clinical variables.

**Table 1:** Patient data collection proforma. CKD, Chronic kidney disease; HCM, Hypertrophic cardiomyopathy; DCM, Dilated cardiomyopathy; RCM, Restrictive cardiomyopathy; ARVC, Arrhythmogenic right ventricular cardiomyopathy; LVH, Left ventricular hypertrophy; EF, Ejection fraction; ACE: Angiotensin-converting enzyme; ICD, Implantable cardioverter defibrillator; CRT, Cardiac resynchronization therapy; PKR, Pakistani rupees.

Section	Variable	Details/Options
1. Demographics	Patient ID	____________
	Name	____________
	Age (years)	____________
	Gender	☐ Male ☐ Female ☐ Other
	BMI (kg/m²)	____________
	Address	____________
	Contact Number	____________
2. Medical History	Duration of Symptoms (months)	____________
	Family History of Cardiomyopathy	☐ Yes ☐ No
	Comorbidities	☐ Hypertension ☐ Diabetes ☐ CKD ☐ None ☐ Other: _____
	History of Smoking	☐ Yes ☐ No
	Alcohol Consumption	☐ Yes ☐ No
	Occupational Exposure to Toxins	☐ Yes ☐ No
3. Cardiomyopathy Diagnosis	Type of Cardiomyopathy	☐ HCM ☐ DCM ☐ RCM ☐ ARVC ☐ Other: ________
	Echocardiographic Findings	☐ LVH ☐ EF%: ____ ☐ Chamber Dilatation ☐ Other: ____
	Ejection Fraction (%)	____________
	NT-proBNP Levels (if available)	____________ pg/mL
	Troponin Levels (if available)	____________ ng/mL
	ECG Abnormalities	☐ Arrhythmia ☐ LVH ☐ Other: _______
4. Socioeconomic & Lifestyle Factors	Education Level	☐ No Education ☐ Primary ☐ Secondary ☐ Higher
	Employment Status	☐ Employed ☐ Unemployed ☐ Retired ☐ Student
	Monthly Income (PKR)	____________
	Dietary Habits	☐ Balanced Diet ☐ High Fat ☐ Low Protein ☐ Other: ____
	Physical Activity	☐ Sedentary ☐ Moderate ☐ Active
5. Treatment & Follow-up	Current Medications	☐ Beta-blockers ☐ ACE inhibitors ☐ Diuretics ☐ Other: ____
	Prior Hospitalizations for Heart Disease	☐ Yes ☐ No
	Cardiac Interventions (e.g., ICD, CRT)	☐ Yes ☐ No
	Follow-up Frequency	☐ Regular ☐ Irregular ☐ None
6. Additional Notes	Clinician’s Comments	____________________________________________________

Statistical analysis

IBM SPSS Statistics for Windows, version 25 (IBM Corp., Armonk, NY) was used for data analysis. Quantitative variables such as age, BMI, and length of illness were expressed as mean ± standard deviation, while qualitative variables-including gender, family history of cardiomyopathy, and cardiomyopathy patterns-were represented as frequencies and percentages. Cardiomyopathy patterns were categorized based on demographic and clinical factors, including family history, age, gender, BMI, and duration of illness.

To control for potential confounding variables, post-stratification analysis was performed using a chi-square test to assess associations between cardiomyopathy patterns and relevant clinical and demographic characteristics. Additionally, subgroup analyses were conducted to identify potential variations in disease presentation across different population segments. A p-value of less than 0.05 was considered statistically significant, ensuring that observed associations were not due to random variation.

Ethical approval

The Northwest General Hospital & Research Centre's research review board in Peshawar granted ethical permission (Ref No: IRB&EC-AHL-4036-2022, dated July 19, 2022). Prior to data collection, all participants gave their informed permission, guaranteeing their privacy and voluntary involvement.

## Results

The majority of participants (32 of 79; 40.51%) were between 51 and 60 years old, with a mean age of 45.72 ± 2.45 years. Among the participants, there were more male individuals (50 of 79; 63.29%) than female individuals (29 of 79; 36.71%). Over one-third of participants (26 of 79; 32.91%) were underweight, while the remaining participants were distributed across normal (18 of 79; 22.78%), overweight (20 of 79; 25.32%), and obese (15 of 79; 18.99%) BMI ranges. Most participants (55 of 79; 69.62%) had experienced symptoms for one month or less. A family history of cardiomyopathy was present in 30 of 79 participants (37.97%; Table 2).

**Table 2:** Demographic and clinical characteristics of study participants.

Category	Subcategory	Frequency	Percent
Age Distribution	16-30 Years	12	15.19
	31-40 Years	19	24.05
	41-50 Years	16	20.25
	51-60 Years	32	40.51
	Mean Age	45.72 ± 2.45	
Gender Distribution	Male	50	63.29
	Female	29	36.71
BMI Classification	Underweight Range	26	32.91
	Normal Range	18	22.78
	Overweight Range	20	25.32
	Obese Range	15	18.99
Duration of Complaints	≤ 1 Month	55	69.62
	> 1 Month	24	30.38
Family History	Yes	30	37.97
	No	49	62.03

**Figure 1:**
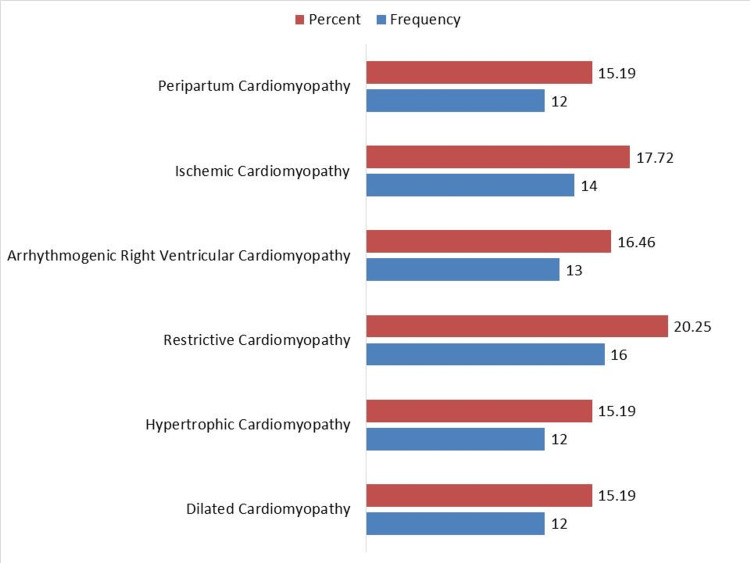
Pattern of cardiomyopathy.

Restrictive cardiomyopathy was the most common type among research participants, accounting for (n=16; 20.25%) of cases. Ischemic cardiomyopathy followed at (n=14; 17.72%), while arrhythmogenic right ventricular cardiomyopathy was observed in (n=13; 16.46%). Peripartum cardiomyopathy, hypertrophic cardiomyopathy, and dilated cardiomyopathy were each present in (n=12; 15.19%) of the individuals. This distribution, as shown in Figure 1, highlights the variety of cardiomyopathy types encountered at the tertiary care hospital, emphasizing the importance of precise diagnosis and individualized treatment plans for affected individuals.

Dilated cardiomyopathy was most prevalent in the 16-30 age group (6 of 12 participants; 50.0%), followed by the 31-40 (3 of 12; 25.0%) and 51-60 (3 of 12; 25.0%) age groups. Hypertrophic cardiomyopathy was equally distributed between the 16-30 (6 of 12; 50.0%) and 31-40 (6 of 12; 50.0%) age groups. Restrictive cardiomyopathy was more common in older individuals, with equal prevalence in the 41-50 (8 of 16; 50.0%) and 51-60 (8 of 16; 50.0%) age groups. The majority of those with arrhythmogenic right ventricular cardiomyopathy (8 of 13; 61.5%) were in the 51-60 age range. Ischemic cardiomyopathy was predominantly seen between 41 and 60 years, with equal distribution in the 41-50 (7 of 14; 50.0%) and 51-60 (7 of 14; 50.0%) age groups. Similarly, peripartum cardiomyopathy was more prevalent among those aged 41-60 (6 of 12; 50.0%; Table 3).

**Table 3:** Age-wise stratification of cardiomyopathy patterns in study participants. df, Degrees of freedom; x², Chi-square.

Pattern of Cardiomyopathy	16-30 Years	31-40 Years	41-50 Years	51-60 Years	Total	Effect Size	df	x^2^	p-value
Dilated Cardiomyopathy	6 (50.0%)	3 (25.0%)	0 (0.0%)	3 (25.0%)	12	0.55	15	71.09	<0.001
Hypertrophic Cardiomyopathy	6 (50.0%)	6 (50.0%)	0 (0.0%)	0 (0.0%)	12				
Restrictive Cardiomyopathy	0 (0.0%)	0 (0.0%)	8 (50.0%)	8 (50.0%)	16				
Arrhythmogenic Right Ventricular Cardiomyopathy	0 (0.0%)	2 (15.4%)	3 (23.1%)	8 (61.5%)	13				
Ischemic Cardiomyopathy	0 (0.0%)	0 (0.0%)	7 (50.0%)	7 (50.0%)	14				
Peripartum Cardiomyopathy	0 (0.0%)	0 (0.0%)	6 (50.0%)	6 (50.0%)	12				

Regarding gender distribution, dilated cardiomyopathy was equally observed in both male individuals (n=6; 50.0%) and female individuals (n=6; 50.0%). Hypertrophic cardiomyopathy was significantly more common in male individuals (n=11; 91.7%) than in female individuals (n=1; 8.3%). Restrictive cardiomyopathy had an equal distribution among male individuals (n=8; 50.0%) and female individuals (n=8; 50.0%). Arrhythmogenic right ventricular cardiomyopathy was more prevalent in male individuals (n=10; 76.9%) compared to female individuals (n=3; 23.1%). Similarly, ischemic cardiomyopathy was more frequent in male individuals (n=10; 71.4%) than in female individuals (n=4; 28.6%). Peripartum cardiomyopathy, however, was exclusively observed in female individuals (n=12; 100.0%; Table 4).

**Table 4:** Gender-based distribution of cardiomyopathy patterns in study participants. df, Degrees of freedom; x², Chi-square.

Pattern of Cardiomyopathy	Male	Female	Total	Effect Size	df	x^2^	p-value
Dilated Cardiomyopathy	6 (50.0%)	6 (50.0%)	12	0.57	5	25.64	<0.001
Hypertrophic Cardiomyopathy	11 (91.7%)	1 (8.3%)	12				
Restrictive Cardiomyopathy	8 (50.0%)	8 (50.0%)	16				
Arrhythmogenic Right Ventricular Cardiomyopathy	10 (76.9%)	3 (23.1%)	13				
Ischemic Cardiomyopathy	10 (71.4%)	4 (28.6%)	14				
Peripartum Cardiomyopathy	0 (0%)	12 (100%)	12				

The BMI-based classification revealed that dilated cardiomyopathy was more frequent in the underweight (n=6; 50.0%) and overweight (n=6; 50.0%) categories, with no cases reported in the normal or obese BMI ranges. Hypertrophic cardiomyopathy followed a similar pattern, with (n=6; 50.0%) in the underweight and (n=6; 50.0%) in the overweight categories. Restrictive cardiomyopathy showed more variation, with (n=4; 25.0%) in the underweight category, (n=4; 25.0%) in the normal range, (n=1; 6.2%) in the overweight range, and the highest percentage in the obese range (n=7; 43.8%). Arrhythmogenic right ventricular cardiomyopathy was predominantly observed in individuals with normal BMI (n=8; 61.5%) and obese BMI (n=5; 38.5%). Ischemic cardiomyopathy was most common in the underweight category (n=7; 50.0%), followed by the normal BMI range (n=6; 42.9%). Peripartum cardiomyopathy cases were most prevalent in the overweight category (n=6; 50.0%), followed by the obese category (n=3; 25.0%; Table 5).

**Table 5:** BMI-based stratification of cardiomyopathy patterns in study participants. df, Degrees of freedom; x², Chi-square.

Pattern of Cardiomyopathy	Underweight Range	Normal Range	Overweight Range	Obese Range	Total	Effect Size	df	x^2^	p-value
Dilated Cardiomyopathy	6 (50.0%)	0 (0.0%)	6 (50.0%)	0 (0.0%)	12	0.51	15	42.83	0.0002
Hypertrophic Cardiomyopathy	6 (50.0%)	0 (0.0%)	6 (50.0%)	0 (0.0%)	12				
Restrictive Cardiomyopathy	4 (25.0%)	4 (25.0%)	1 (6.2%)	7 (43.8%)	16				
Arrhythmogenic Right Ventricular Cardiomyopathy	0 (0.0%)	8 (61.5%)	0 (0.0%)	5 (38.5%)	13				
Ischemic Cardiomyopathy	7 (50.0%)	6 (42.9%)	1 (7.1%)	0 (0.0%)	14				
Peripartum Cardiomyopathy	3 (25.0%)	0 (0.0%)	6 (50.0%)	3 (25.0%)	12				

In terms of duration of symptoms, dilated cardiomyopathy and hypertrophic cardiomyopathy were exclusively found in individuals who presented within ≤1 month (n=12; 100.0%). Restrictive cardiomyopathy had an equal distribution among those presenting ≤1 month (n=8; 50.0%) and those presenting >1 month (n=8; 50.0%). Arrhythmogenic right ventricular cardiomyopathy was mostly observed in those who reported symptoms >1 month (n=12; 92.3%). Similarly, ischemic cardiomyopathy was more common in those presenting within ≤1 month (n=10; 71.4%) than those with symptoms lasting >1 month (n=4; 28.6%). Peripartum cardiomyopathy was exclusively observed in those who presented within ≤1 month (n=12; 100.0%; Table 6).

**Table 6:** Duration of complaints and its association with cardiomyopathy patterns. df, Degrees of freedom; x², Chi-square.

Pattern of Cardiomyopathy	≤ 1 Month	> 1 Month	Total	Effect Size	df	x^2^	p-value
Dilated Cardiomyopathy	12 (100.0%)	0 (0.0%)	12	0.58	5	38.47	<0.001
Hypertrophic Cardiomyopathy	12 (100.0%)	0 (0.0%)	12				
Restrictive Cardiomyopathy	8 (50.0%)	8 (50.0%)	16				
Arrhythmogenic Right Ventricular Cardiomyopathy	1 (7.7%)	12 (92.3%)	13				
Ischemic Cardiomyopathy	10 (71.4%)	4 (28.6%)	14				
Peripartum Cardiomyopathy	12 (100.0%)	0 (0.0%)	12				

The impact of family history on cardiomyopathy prevalence revealed that dilated cardiomyopathy was more common in individuals with a positive family history (n=9; 75.0%) compared to those without (n=3; 25.0%). Hypertrophic cardiomyopathy was evenly distributed between those with (n=6; 50.0%) and without (n=6; 50.0%) a family history. Restrictive cardiomyopathy was mostly observed in those without a family history (n=15; 93.8%), while only (n=1; 6.2%) had a positive family history. Arrhythmogenic right ventricular cardiomyopathy followed a similar trend, with (n=12; 92.3%) of cases occurring in those without a family history. Ischemic cardiomyopathy was more prevalent in those without a family history (n=10; 71.4%) than those with one (n=4; 28.6%). Peripartum cardiomyopathy was strongly associated with a positive family history, with (n=9; 75.0%) of cases reported among those with a familial connection (Table 7).

**Table 7:** Impact of family history on cardiomyopathy patterns in study participants. df, Degrees of freedom; x², Chi-square.

Pattern of Cardiomyopathy	Yes	No	Total	Effect Size	df	x^2^	p-value
Dilated Cardiomyopathy	9 (75.0%)	3 (25.0%)	12	0.59	5	27.13	0.00005
Hypertrophic Cardiomyopathy	6 (50.0%)	6 (50.0%)	12				
Restrictive Cardiomyopathy	1 (6.2%)	15 (93.8%)	16				
Arrhythmogenic Right Ventricular Cardiomyopathy	1 (7.7%)	12 (92.3%)	13				
Ischemic Cardiomyopathy	4 (28.6%)	10 (71.4%)	14				
Peripartum Cardiomyopathy	9 (75.0%)	3 (25.0%)	12				

## Discussion

Cardiomyopathy is a collection of myocardial conditions that have a major impact on cardiac function and may result in arrhythmias, heart failure, and other problems. Improving diagnosis and therapy requires an understanding of the incidence and patterns of various forms of cardiomyopathy. Emerging evidence suggests that cardiac conditions, including arrhythmias and congestive heart failure, not only lead to hemodynamic compromise but may also contribute to cognitive decline [18]. The objective of this study was to investigate the patterns of cardiomyopathy in patients presenting to a tertiary care hospital in Peshawar, Pakistan, and to analyze their distribution across demographic and clinical factors. This study aimed to assess whether these patterns align with global trends and to explore potential genetic and environmental contributions. Additionally, we examined the association between cardiomyopathy subtypes and key variables such as age, gender, BMI, family history, and duration of illness. Understanding these patterns could help improve early diagnosis and management strategies tailored to the local population.

With 20.25% of patients, restrictive cardiomyopathy was the most common condition in our research. This result is consistent with previous research in low-income regions, where restrictive cardiomyopathy is frequently linked to pericardial diseases, nutritional deficiencies, and infectious etiologies such as tuberculosis and amyloidosis. Additionally, socioeconomic determinants, including limited access to healthcare, delayed diagnosis, and higher exposure to environmental risk factors, may contribute to the observed patterns. A study by Rapezzi et al. (2022) identified restrictive cardiomyopathy as the predominant subtype of heart failure in similar settings [19]. Furthermore, the impact of social determinants of health on cardiovascular outcomes, suggests that factors such as food insecurity, inadequate healthcare access, and socioeconomic disparities may further influence the prevalence and progression of restrictive cardiomyopathy [10].

In 15.19% of the patients, dilated cardiomyopathy (DCM) and hypertrophic cardiomyopathy (HCM) were found. Although DCM is usually seen in older persons, 50% of the participants in our research were between the ages of 16 and 30. Our population's early start is in line with other research that found groups with genetic predispositions had greater rates of early-onset DCM [20]. HCM, on the other hand, had a more evenly distributed age distribution, with 50% of cases falling into the 16-30 and 31-40 age categories. Due to its family inheritance patterns, HCM is often detected in early adulthood [21,22].

Our study's gender distribution revealed a male preponderance for arrhythmogenic right ventricular cardiomyopathy (76.9%) and hypertrophic cardiomyopathy (HCM) (91.7%), which aligns with existing literature suggesting that both conditions are more frequently diagnosed in men [23]. However, arrhythmogenic right ventricular cardiomyopathy may be underdiagnosed in women due to less frequent cardiovascular screening and differences in clinical presentation. As noted by Lakdawala et al. (2021), genetic variations in sarcomere genes contribute to a higher HCM risk in male individuals [24]. Additionally, regional factors such as late presentation, limited access to specialized diagnostic tools, and nutritional deficiencies could influence cardiomyopathy patterns in this population.

On the other hand, peripartum cardiomyopathy was exclusively observed in women (58.3%), consistent with prior studies emphasizing the role of hormonal fluctuations and pregnancy-related hemodynamic stress in the pathogenesis of this condition [25]. According to our study's BMI analysis, underweight and overweight categories were substantially correlated with both DCM and HCM (p = 0.000). This finding is consistent with earlier studies that show a higher risk of heart disease associated with extremes in body mass index [26].

According to research by Ren et al. (2021), underweight and obesity are risk factors for a number of cardiomyopathies, such as DCM and HCM [27]. Lastly, 75% of patients in both groups had a family history of DCM and peripartum cardiomyopathy, indicating a substantial correlation between the two conditions. This adds credence to the mounting evidence that these cardiomyopathies are influenced by hereditary factors. Numerous investigations have shown that genetic mutations in sarcomeric proteins have a role in the development of familial DCM, which is particularly well-documented in the literature [28].

The findings of this research provide important insight into the prevalence and epidemiology of various cardiomyopathies in our community. In order to enhance early detection and management, several targeted strategies could be implemented. First, enhanced screening programs should be established, particularly for at-risk populations such as women, individuals with a family history of cardiomyopathy, and those with metabolic disorders. Second, genetic counseling and testing could help identify hereditary cardiomyopathies at an earlier stage, allowing for proactive interventions. Third, improving healthcare accessibility through community-based clinics and outreach programs can facilitate timely diagnosis and reduce late presentations. Fourth, public health awareness campaigns focusing on modifiable risk factors, including diet, exercise, and infection prevention, may help mitigate disease progression. Finally, integrating regional epidemiological data into healthcare policies can aid in developing patient-specific management strategies, ultimately improving outcomes.

Study strengths and limitations

This study's strength lies in its comprehensive approach to examining the patterns of cardiomyopathy across a diverse patient population, with a focus on both clinical and demographic characteristics. By including multiple cardiomyopathy subtypes, the study provides valuable insights into the distribution of the disease within the local healthcare system. However, several limitations must be acknowledged.

First, the cross-sectional design restricts the ability to establish causal relationships, and the lack of longitudinal follow-up prevents an assessment of disease progression over time. Second, the study does not account for key socioeconomic factors, such as education level, income, dietary habits, and environmental exposures, all of which can significantly influence cardiomyopathy prevalence and outcomes. Third, the tertiary care setting may introduce selection bias, as patients with more severe or complex cases are more likely to be included, potentially overestimating the prevalence of advanced cardiomyopathy compared to the general population. Fourth, the absence of genetic and molecular data limits the ability to explore hereditary risk factors that contribute to disease development. Additionally, the lack of cardiac biomarker analysis (e.g., NT-proBNP, troponins) restricts insights into the severity and etiology of cardiomyopathies, which could have enhanced diagnostic precision.

Future research should incorporate longitudinal studies, a broader and more diverse patient population, and biomarker-based assessments to provide a more comprehensive understanding of cardiomyopathy patterns and their determinants.

## Conclusions

This research highlights restrictive and ischemic cardiomyopathy as the most prevalent subtypes in the local population and offers important insights into the incidence and distribution of different cardiomyopathy patterns in a tertiary care hospital. In addressing cardiomyopathy, the results highlight the value of early diagnosis while taking into account variables including age, gender, BMI, length of complaints, and family history. Although the study provides valuable information on illness patterns, further research is required to fully understand the underlying reasons and enhance therapeutic care approaches for individuals with cardiomyopathy. This includes bigger sample sizes and genetic studies.

## References

[1] Maron BJ, Towbin JA, Thiene G (2006). Contemporary definitions and classification of the cardiomyopathies: an American Heart Association scientific statement from the Council on Clinical Cardiology, Heart Failure and Transplantation Committee; Quality of Care and Outcomes Research and Functional Genomics and Translational Biology Interdisciplinary Working Groups; and Council on Epidemiology and Prevention. Circulation.

[2] Kuriachan VP, Sumner GL, Mitchell LB (2015). Sudden cardiac death. Curr Probl Cardiol.

[3] Ciarambino T, Menna G, Sansone G, Giordano M (2021). Cardiomyopathies: an Overview. Int J Mol Sci.

[4] Bozkurt B, Colvin M, Cook J (2016). Current diagnostic and treatment strategies for specific dilated cardiomyopathies: a scientific statement from the American Heart Association. Circulation.

[5] Pasqualucci D, Iacovoni A, Palmieri V (2022). Epidemiology of cardiomyopathies: essential context knowledge for a tailored clinical work-up. Eur J Prev Cardiol.

[6] Smail M, Rupee K, Rupee S (2024). Understanding cardiomyopathy: epidemiology, risk factors. Etiology, prevention and management of cardiomyopathy. Intechopen.

[7] Finocchiaro G, Magavern E, Sinagra G (2017). Impact of demographic features, lifestyle, and comorbidities on the clinical expression of hypertrophic cardiomyopathy. J Am Heart Assoc.

[8] Pearson TA (1999). Cardiovascular disease in developing countries: myths, realities, and opportunities. Cardiovasc Drugs Ther.

[9] Efthimiadis GK, Parcharidou D, Pagourelias ED (2010). Prevalence and clinical outcomes of incidentally diagnosed hypertrophic cardiomyopathy. Am J Cardiol.

[10] Borkowski P, Borkowska N, Mangeshkar S, Adal BH, Singh N (2024). Racial and socioeconomic determinants of cardiovascular health: a comprehensive review. Cureus.

[11] Patil VC, Desai N, Galande C (2014). Clinical and echocardiogram profile of cardiomyopathy at tertiary care centre. J Cardiovasc Dis Res.

[12] Wijeysundera HC, Trubiani G, Wang X (2013). A population-based study to evaluate the effectiveness of multidisciplinary heart failure clinics and identify important service components. Circ Heart Fail.

[13] Tesfaye S, Shifeta M, Hirigo AT (2020). Pattern of cardiac diseases and co-existing morbidities among newly registered cardiac patients in an adult cardiac referral clinic of Hawassa University Comprehensive Specialized Hospital, Southern-Ethiopia. Vasc Health Risk Manag.

[14] Khera R, Pandey A, Ayers CR (2017). Contemporary epidemiology of heart failure in fee-for-service Medicare beneficiaries across healthcare settings. Circ Heart Fail.

[15] Olawade DB, Aderinto N, Olatunji G, Kokori E, David-Olawade AC, Hadi M (2024). Advancements and applications of artificial intelligence in cardiology: current trends and future prospects. J Med Surg Public Health.

[16] Maron BJ, Desai MY, Nishimura RA (2022). Diagnosis and evaluation of hypertrophic cardiomyopathy: JACC state-of-the-art review. J Am Coll Cardiol.

[17] Martinez HR, Beasley GS, Miller N, Goldberg JF, Jefferies JL (2021). Clinical insights into heritable cardiomyopathies. Front Genet.

[18] Varrias D, Saralidze T, Borkowski P, Pargaonkar S, Spanos M, Bazoukis G, Kokkinidis D (2024). Atrial fibrillation and dementia: pathophysiological mechanisms and clinical implications. Biomolecules.

[19] Rapezzi C, Aimo A, Barison A (2022). Restrictive cardiomyopathy: definition and diagnosis. Eur Heart J.

[20] Rich MW (1997). Epidemiology, pathophysiology, and etiology of congestive heart failure in older adults. J Am Geriatr Soc.

[21] McNally EM, Mestroni L (2017). Dilated cardiomyopathy: genetic determinants and mechanisms. Circ Res.

[22] Marian AJ, Braunwald E (2017). Hypertrophic cardiomyopathy: genetics, pathogenesis, clinical manifestations, diagnosis, and therapy. Circ Res.

[23] Preveden A, Golubovic M, Bjelobrk M (2022). Gender related differences in the clinical presentation of hypertrophic cardiomyopathy-an analysis from the SILICOFCM database. Medicina (Kaunas).

[24] Lakdawala NK, Olivotto I, Day SM (2021). Associations between female sex, sarcomere variants, and clinical outcomes in hypertrophic cardiomyopathy. Circ Genom Precis Med.

[25] Mielniczuk LM, Williams K, Davis DR (2006). Frequency of peripartum cardiomyopathy. Am J Cardiol.

[26] Kenchaiah S, Evans JC, Levy D (2002). Obesity and the risk of heart failure. N Engl J Med.

[27] Ren J, Wu NN, Wang S, Sowers JR, Zhang Y (2021). Obesity cardiomyopathy: evidence, mechanisms, and therapeutic implications. Physiol Rev.

[28] Niimura H, Patton KK, McKenna WJ, Soults J, Maron BJ, Seidman JG, Seidman CE (2002). Sarcomere protein gene mutations in hypertrophic cardiomyopathy of the elderly. Circulation.

